# Entanglement beating in free space through spin–orbit coupling

**DOI:** 10.1038/lsa.2018.9

**Published:** 2018-05-04

**Authors:** Eileen Otte, Carmelo Rosales-Guzmán, Bienvenu Ndagano, Cornelia Denz, Andrew Forbes

**Affiliations:** 1 Institute of Applied Physics, University of Muenster, Muenster D-48149, Germany; 2 School of Physics, University of the Witwatersrand, Wits 2050, South Africa

**Keywords:** classical entanglement, complex light fields, entanglement oscillation, spin–orbit coupling

## Abstract

It is well known that the entanglement of a quantum state is invariant under local unitary transformations. This rule dictates, for example, that the entanglement of internal degrees of freedom of a photon remains invariant during free-space propagation. Here, we outline a scenario in which this paradigm does not hold. Using local Bell states engineered from classical vector vortex beams with non-separable degrees of freedom, the so-called classically entangled states, we demonstrate that the entanglement evolves during propagation, oscillating between maximally entangled (purely vector) and product states (purely scalar). We outline the spin–orbit interaction behind these novel propagation dynamics and confirm the results experimentally, demonstrating spin–orbit coupling in paraxial beams. This demonstration highlights a hitherto unnoticed property of classical entanglement and simultaneously offers a device for the on-demand delivery of vector states to targets, for example, for dynamic laser materials processing, switchable resolution within stimulated emission depletion (STED) systems, and a tractor beam for entanglement.

## Introduction

Under local unitary operations, for example, when propagating through a unitary channel, the degree of entanglement does not change. This finding is true for both non-local entanglement, that is, light fields (including single photon, multi-photons or coherent light) that simultaneously exist in physically separated locations, and for local entanglement, that is, between the internal degrees of freedom of a single photon. Recently, it has become of interest to study the latter and to mimic the former using vector states of classical light^1, 2, 3, 4, 5, 6, 7, 8, 9, 10^. This approach is possible because the central feature of entanglement, non-separability, is not limited to quantum systems: classical vector beams are likewise non-separable, for example, in their polarization and spatial modes. However, whether such fields can be called ‘classically entangled’ is an open question^1, 7^, in practice, this property has been exploited for real-time quantum error correction^11^, communication^12, 13, 14, 15, 16^, laser materials processing^17, 18, 19^ and metrology^20, 21, 22^. In addition, in imaging^23, 24, 25, 26^, where tightly focused radially polarized fields are known to produce the narrowest spot size^27, 28, 29, 30^, classically entangled light fields allow super-resolution microscopy techniques^31, 32^.

Here, we demonstrate that entanglement can evolve during propagation in free space using classically entangled vector vortex beams, which are non-separable in orbital and spin angular momentum. We engineer superpositions of these beams to prove the dynamic change of entanglement upon propagation through spin–orbit (SO) coupling. Such SO coupling^33^ has been observed through the spin-Hall effect of light at planar interfaces, by non-paraxial light (tightly focused by high numerical aperture lenses), and with paraxial light in anisotropic and inhomogeneous structures, for example, using geometric phase^34^. Here, we show that it is possible with paraxial light in free space. Through this SO coupling, we demonstrate entanglement beating from fully entangled (completely non-separable) to no entanglement (fully separable), and by a phase adjustment, we evince the possible transport of entanglement, which is reminiscent of tractor beams for particle transport^35, 36, 37, 38^. This realization may open new avenues in quantum and classical communication as well as in improved materials processing (where vector beams and scalar polarized beams are crucial) and enhanced switchable imaging in stimulated emission depletion (STED) microscopy.

## Materials and methods

### Concept

Consider a vector beam that is composed of a superposition of two orthogonally polarized Laguerre–Gaussian modes 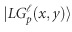 given by^9^


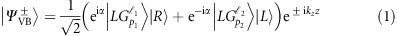


where we assume a propagation in the ±*z*-direction, approximated by the factor 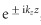, where 
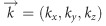
 is the wave vector expressed in terms of the wavelength *λ*, as *k*=2*π*/*λ*. The kets 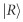 and 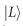 represent the unit vector of right- and left-handed circular polarization states, respectively, and *α* defines the phase relation between the two states. The indices 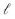 and *p* denote the azimuthal and radial degrees of freedom, respectively, the former being related to the orbital angular momentum (OAM) of the Laguerre–Gaussian (LG) beam. In the following description, we will restrict ourselves to the case in which 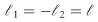 and *p*_1_=*p*_2_=*p*, but it can be extended to other cases. Equation (1) can be conveniently written as^39^


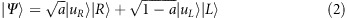


where 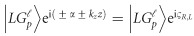 are represented by the ket 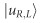 and the relative weightings of 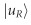 and 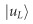 by *a*. Moreover, 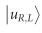 satisfies the normalization condition 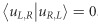.

The degree of non-separability (classical entanglement) 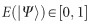 of a vector field as defined by Equation (2) can be computed using tools from quantum mechanics. Here, we consider the entanglement entropy, originally derived for quantum states^40, 41^ and later extended to classical non-separable states^39^ as


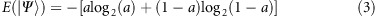


Consequently, if we analyze a vector beam 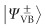 under a unitary transformation, that is, propagation in free space along the ±*z*-direction (Equation (1)), where *a*=1/2 for all *z* values, we observe a spatially invariant degree of entanglement 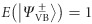.

**Figure 1:**
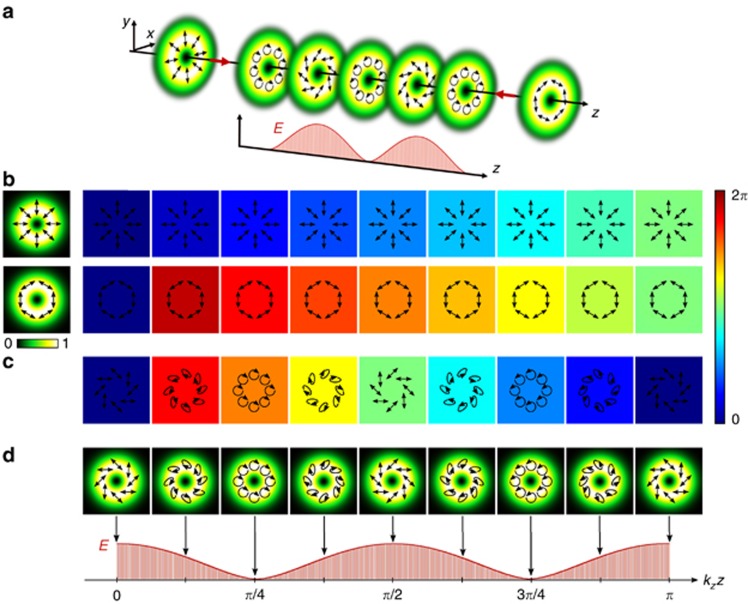
Schematic representation of the investigated field with a *z*-dependent degree of entanglement. (**a**) Concept, (**b**) phase change of the radial/azimuthal beam (top/bottom) relative to the initial phase, (**c**) absolute value of the relative phase difference between the radial and azimuthal beam, (**d**) change in polarization upon intensity (top) with the corresponding degree of entanglement *E* (bottom) for superimposed counter-propagating radial and azimuthal vector beams, all depending on the propagation distance *z* (*k*_*z*_*z*∈[0,*π*]). Further, **b** and **c** include the respective polarization distributions per distance.

Remarkably, we can engineer a light field 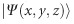 with a *z*-dependent degree of entanglement 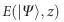 by combining two orthogonal vector beams 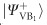 and 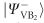, coaxially propagating in opposite directions, as illustrated in Figure 1a. For example, these orthogonal fields can be generated by setting 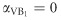 and 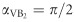 in Equation (1), namely,


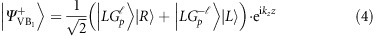


and


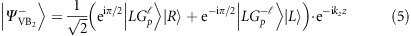


with a phase distribution as a function of *z* as shown in Figure 1b, top and bottom, respectively, for the case 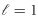, *p*=0. The normalized field that results from such a superposition takes the form


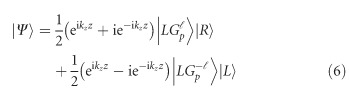


whose polarization evolution upon propagation for the regarded example is shown in Figure 1c and 1d, respectively. Here, Figure 1c includes the change in the relative phase between the superimposed beams, which represents the origin of the resultant *z*-variant polarization structure. The new engineered state 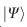 undergoes an oscillatory transition between fully vector and fully scalar modes of light, which can be described by the SO interaction^33^. The total angular momentum of our field always satisfies *J*_*z*_=0 (for superposition beams constructed from the 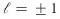 subspace) but with oscillatory spin and orbit components that vary as *S*_*z*_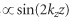 and 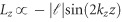, respectively. As a result of the out-of-phase oscillation for opposite helicities, as the OAM component increases, the spin component decreases concomitantly to conserve the total angular momentum (see Supplementary Information).

This variation between the scalar and vector modes manifests itself through a change in the degree of entanglement, as defined by Equation (3), which for the new light field 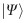 takes the form


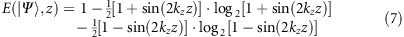


(details with respect to the calculations can be found within the Supplementary Information). Thus, the state undergoes a periodic variation in the degree of entanglement as a function of *z*, as illustrated in Figure 1d, bottom, while the intensity profile remains constant. Full entanglement, that is, maximal non-separability 
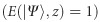
 is achieved at *z*=*nλ*/4, 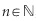, whereas non-entanglement, that is, complete separability 
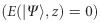
 is observed at *z*=(2*n*+1)*λ*/8, 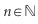. Note that space-variant entanglement of the form 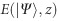 can be realized by any OAM subspace 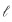 by the superposition of orthogonal vector fields 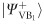 and 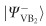, as long as they carry the same radial order *p*_1,2_(VB_1,2_)=*p*. In contrast, if we superimpose two counter-propagating scalar modes of opposite helicity and orthogonal polarization, the degree of entanglement will remain constant (see Supplementary Information).

This unique property of the field 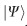 provides a means to facilitate the transport of a chosen degree of entanglement across arbitrary distances, by simply applying a phase adjustment *ϕ*, which is reminiscent of tractor beams^35, 36, 37, 38^. To illustrate this approach, we can replace the propagation factor in Equation (1) by the factor 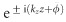. In this way, the maximum degree of entanglement 
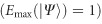
, for example, can be transported to a position 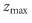 according to the expression


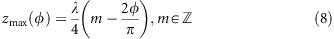


This means that any chosen state can be conveyed to a specific position in space, along the propagation axis, by simply adjusting the phase *ϕ*. Moreover, by applying a time-dependent phase shift *ϕ*(*t*), it is possible to impart a time-dependent movement of a regarded maximum with an axial velocity given by


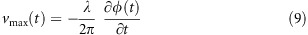


### Experimental details

**Figure 2:**
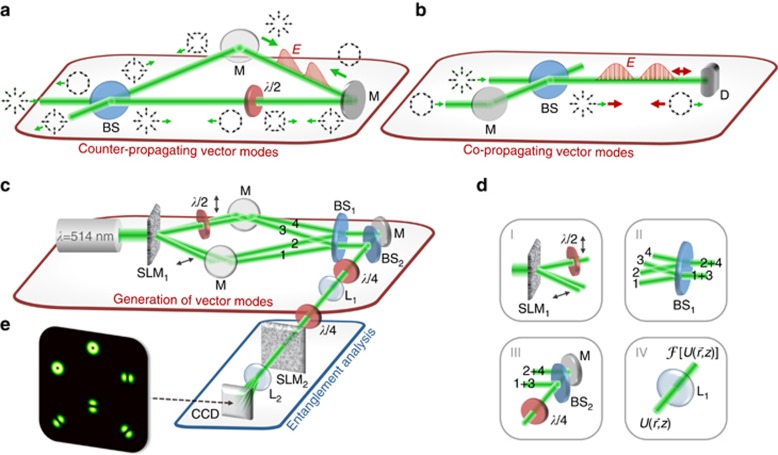
Sketch of the experimental concept: approach of (**a**) counter- and (**b**) co-propagating vector modes for the realization/investigation of the light field 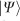. (**c**) Applied system for generation (red box) and analysis (blue box, (**e**)) of 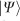 with experimental steps indicated in (**d**). *λ*/2, half-wave plate; *λ*/4, quarter-wave plate; BS_1,2_, beam splitter; CCD, camera; L_1,2_, lens; M, mirror; SLM_1,2_, spatial light modulator.

A simple method to generate a light field 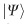 with local entanglement beating is via an interferometric approach. An exemplary system is sketched in Figure 2a. By combining a Sagnac interferometer with a half-wave plate (diagonally oriented), a single incident vector beam, for example, radially polarized, can be used for the generation of a standing wave according to Equations (4), (5), (6), whose local degree of entanglement *E* oscillates upon propagation. Note that in each arm of the interferometer, counter-propagating (green arrows) vector modes of orthogonal polarization will give rise to a classically entangled standing wave, as indicated by a red curve in one of the arms.

Even if this approach of counter-propagating beams is very intuitive, the investigation of the light field 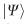 would be challenging because any measuring device inserted in the path would destroy the oscillatory behavior of the light field 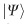. Here, we propose an alternative approach that allows us to quantify the spatially varying degree of entanglement. This approach is based on the engineered superposition of co-propagating orthogonally polarized vector modes, as visualized in Figure 2b. By applying digital propagation, we can artificially counter-propagate the two modes (red arrows), which physically co-propagate in the same direction (green arrows), enabling us to investigate the light field 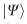 along the beam path.

The digital propagation of a light field 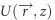 propagating in the *z*-direction is based on the angular spectrum^42, 43^, according to which 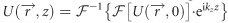, where 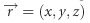 are the coordinates in real space, and 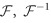 are the Fourier and inverse Fourier transforms, respectively. Next, by the application of Fourier holograms in combination with a phase shift ±*k*_*z*_*z*, encoded on a spatial light modulator (SLM), we were able to digitally propagate a light field in the ±*z*-direction. To independently control the phase shift of each vector mode, for the artificial generation of counter-propagating vector modes, we developed a new method that facilitates the generation of any vector beam using a multiplexing approach enabled by an SLM^44^. This method allows not only simultaneous generation of multiple vector modes, but also their independent manipulation, such as digital propagation.

The idea behind our method is to encode a superposition of different holograms, each with a different spatial carrier frequency (blazed grating), on a single SLM. Thus, each beam is sent to different transverse positions in space, which allows manipulation of their polarization independently, as required for vector beam generation. For example, to generate a radially polarized vector beam, we multiplexed the corresponding holograms to create two helical LG beams with opposite topological charges 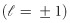 on the SLM. A half-wave plate placed in the path of one beam changes its polarization from horizontal to vertical. Both beams were then recombined and passed through a quarter-wave plate to change the horizontal and vertical polarizations into left- and right-circular polarizations, respectively, thus generating the desired vector beam^45^.

In the present case, where we realized a superposition of two cylindrical vector beams VB_1,2_ (see Figure 2c, red box, and Figure 2d), four vortex beams were multiplexed in the SLM (SLM_l_; Fourier holograms), manipulated accordingly and (counter-) propagated digitally (Fourier relation between SLM_l_ and SLM_2_ by lens L_1_) to investigate the desired field 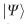 within the observation plane (SLM_2_). In this way, the detection system can remain static while the created vector beams artificially propagate in opposite directions. Beyond this, digital propagation, encoded on the SLM as a phase shift *ϕ*, facilitates the realization of a chosen degree of entanglement at the observation plane, which is similar to the case of tractor beams.

### Theory of entanglement entropy

For the analysis of the light field 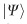, we determined the degree of classical entanglement, that is, the degree of non-separability, in different (*x*, *y*)-planes. An appropriate tool for this concern is the quantum mechanics entanglement entropy^39, 40^


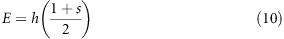


with 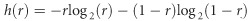. Here, *s* is the length of the Bloch vector, given by 
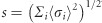
 with *i*={1, 2, 3}, where 〈*σ*_*i*_〉 are the expectation values of the Pauli operators. These values are obtained by a set of 12 normalized, on-axis intensity measurements or six identical measurements for two different basis states^39, 41^.

We chose circular polarization as a basis. As a consequence, the projection measurements are given by two modes that carry the OAM of topological charge 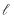 and 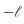, in addition to four superposition states represented by 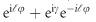 with 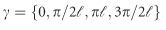 (*φ*: azimuthal angle in polar coordinates). In the case at hand, we investigate the vector modes of first order (cf. Results and Discussion section), and hence, the projection measurements are performed for 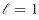.

According to Table 1, the expectation values 〈*σ*_*i*_〉 are calculated from


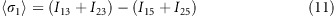



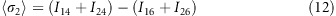



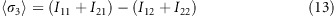


To determine the entanglement entropy *E* experimentally, we measure the on-axis intensity values *I*_*uv*_ with *u*, *v*∈{1, 2, 3}, as indicated in Figure 2c, blue box, and Figure 2e. Therefore, polarization projections are performed by the use of a quarter-wave plate (*λ*/4) set to ±45° in combination with a polarization-sensitive spatial light modulator (SLM_2_) and OAM projections by a phase pattern on this modulator. The respective phase pattern carries the information of all six OAM projections, in which each of them is assigned to another spatial carrier frequency^46^. The application of this demultiplexing hologram results in six outputs on the CCD camera. Figure 2e positioned in Fourier relation with the observation plane (SLM_2_) by a lens (L_2_), which enables a single-shot measurement for each polarization basis.

For the entanglement entropy analysis in different (*x*, *y*)-planes of the light field, artificial propagation in the *z*-direction is applied. Further, the intensities *I*_*uv*_ for different planes are normalized by *I*_11_ + *I*_12_ + *I*_21_ + *I*_22_ for left- and right-circular polarization basis.

## Results and discussion

To verify that the field 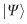 follows the entanglement dynamics predicted by Equation (7), we experimentally generated and superimposed two orthogonal vector beams (according to Equations (4) and (5)), using the setup shown in Figure 2c, as indicated by the red box. For simplicity but without the loss of generality, we chose first-order radially and azimuthally polarized modes with 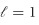 and *p*=0. Magnifications of the different sections of the generation process are shown in Figure 2d. The desired light field 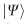 for a specific *z*-position is realized in the Fourier plane (SLM_2_, observation plane) of SLM_1_.

**Figure 3:**
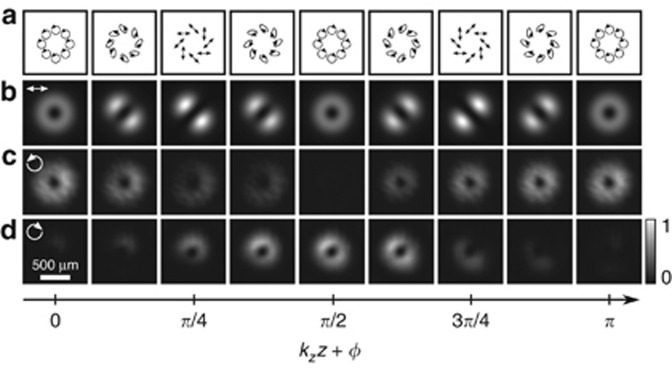
Intensity profile of the investigated light field 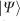 for various *z*-positions in units of *k*_*z*_*z* + *ϕ* (*ϕ*=−*π*/4) with corresponding polarization structure in (**a**). (**b**) Normalized intensity profile of the field 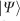, passing through a horizontally aligned polarizer (data from simulation). Experimental results of counter-oscillating intensities for (**c**) 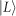 and (**d**) 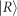 polarization components.

The artificially generated field 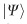 can be separated into its 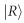 and 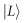 parts, with each including two counter-propagating LG modes of the same helicity. For each polarization, one mode propagates in the +*z*-direction, and the other propagates in the -*z*-direction, which is achieved through digital propagation enabled by SLM_1_. The digital propagation was encoded as 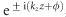, in which we chose *ϕ* to be a discrete phase offset of –*π*/4. Using a CCD camera positioned in the observation plane, we recorded the intensity profile of the 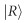 and 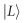 components separately by shutting beams 3 and 4 or 1 and 2 (cf. Figure 2c and 2d), respectively. The results are shown in Figure 3. In Figure 3b, we show the simulated transverse intensity profile of 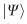 when a horizontally aligned polarizer is positioned in front of the CCD, thus reflecting the polarization distribution illustrated in Figure 3a and Figure 1. The normalized intensity profiles for the 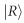 (beam 1+2) and 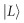 (beam 3+4) polarization components are shown in Figure 3c and 3d, respectively, for the different positions *k*_*z*_*z* + *ϕ*∈[0, *π*] (arrow at the bottom). For both the 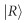 and 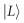 parts, we observe a sinusoidal variation in the intensity that depends on *k*_*z*_*z*+*ϕ*, which represents a longitudinal interference pattern of included beams. Furthermore, the variation in intensity for 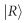 and 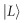 is out of phase, that is, the 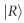 components carry maximum intensity while the 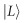 parts are at minimum, and vice versa. This behavior is attributed to the phase shift 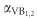, which was used to create orthogonally polarized vector beams (cf. Equation (6)). Moreover, these counter-fluctuating intensities evince the variation between pure vector and pure scalar states for 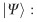 If the 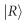 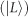 polarized components are at a maximum, while the 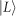 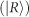 parts disappear, then 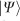 is represented solely by the 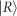 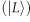 components, and thus, the light field is purely scalar with 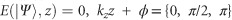. In contrast, if the 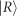 and 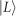 parts are of equal intensity, then 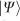 is a pure vector mode with 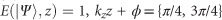. Between these extreme cases, a smooth transition is found (cf. Figure 3b).

### Entanglement oscillation

To quantitatively verify the longitudinal entanglement oscillation of 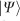, we performed an entanglement entropy analysis while digitally propagating the field. Using this approach, we determined the degree of entanglement 
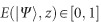
 as a function of *k*_*z*_*z* + *ϕ*. The respective experimental method is visualized in Figure 2c (blue box) and Figure 2e.

**Figure 4:**
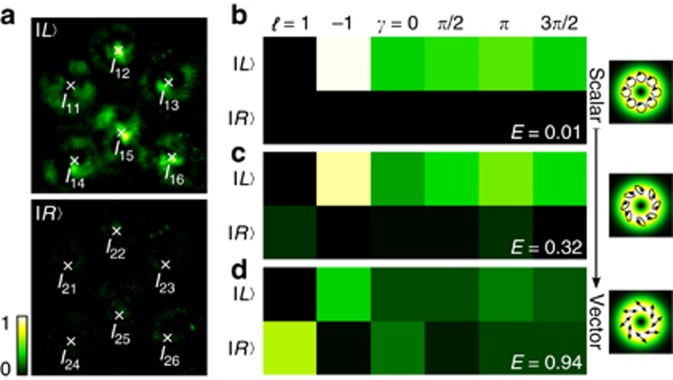
(**a**) Typical intensity images acquired by a CCD camera to determine the degree of entanglement *E* in the case of a scalar beam. The corresponding intensities *I*_*uv*_ with *u*,*v*∈{1, 2, 3}, arranged according to Table 1 for the cases of a (**b**) scalar, (**c**) semi-vector and (**d**) vector beams, with corresponding values of *E*=0.01, 0.32, 0.94.

**Figure 5:**
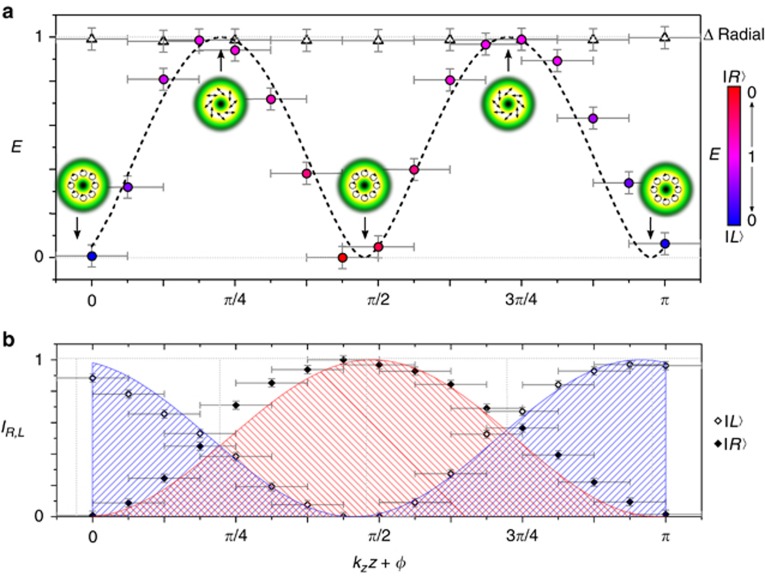
Propagation dynamics of entanglement: (**a**) Entanglement analysis of pure radial vector mode (black triangles) and the light field 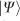. Measured *E* as a function of *k*_*z*_*z* + *ϕ* (*ϕ*=−*π*/4) of the latter is marked by black circles filled according to the ratio between the 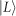 and 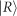 parts (see scale bar). Exemplary modes are shown as green insets. The black dashed curve represents the theoretical fit according to Equation (7). (**b**) Respective intensity *I*_*R*,*L*_ of the 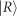 (red fit, black filled diamonds) and 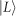 (blue fit, black hollow diamonds) components of 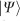, oscillating out of phase.

Figure 4a shows typical intensity images obtained in experiments per *z*-distance and from which *E* is computed. The illustrated case corresponds to the scalar field shown in Figure 4b. Figure 4b–4d shows the intensity values, normalized and arranged in the form of Table 1. Here, we show three cases: scalar, semi-vector and vector beam, with the corresponding values *E*=0.01, 0.32 and 0.94, respectively. The complete set of experimental *E* values obtained as a function of the propagation distance *z* is presented in Figure 5. Here, the degree of entanglement (Figure 5a) and the normalized intensity of the right-/left-handed circularly polarized light *I*_*R*,*L*_ (Figure 5b) are illustrated as a function of *k*_*z*_*z* + *ϕ*. Errors of *k*_*z*_*z* + *ϕ* are given by SLM flickering (±*π*/16), whereby error bars for *E* (±0.05) or *I*_*R*,*L*_ (±0.03) are given by inaccuracies within the experimental method/ system.

For comparison, we experimentally performed an entanglement analysis of a pure radial vector mode (beam 1+3). As theoretically expected (cf. Materials and Methods, Theory), this beam reveals an entanglement entropy of approximately *E*=1 for all propagation distances, as depicted by the black triangles in Figure 5a. In contrast, the entanglement dynamics of the light field 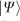 given by Equation (6) confirms our theoretical predictions, oscillating between pure scalar and pure vector, as shown in Figure 5a. The data are represented by black circles filled according to the ratio between the included 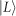 (blue) and 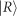 (red) polarized parts (see scale bar). The green insets indicate the modes of light at specific positions. The experimental results reflect the theoretical description in Equation (7) with *k*_*z*_*z* replaced by *k*_*z*_*z* + *ϕ*' perfectly, as illustrated by the corresponding fit in Figure 5a (black dashed curve). The fitting parameter *ϕ*' has a value of −0.71 and, thus, almost matches the chosen setting of *ϕ*=−*π*/4.

Figure 5b shows simultaneously determined counter-fluctuating intensity curves for 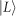 (blue fit, black hollow diamonds) and 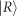 (red fit, black filled diamonds). Obviously, these curves mirror the propagation dynamics of entanglement and the involved variation in the ratio between 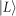 and 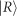, as demonstrated in Figure 5a. A slight shift with respect to the positions of the extrema of 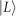 and 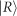 can be observed, which reflects the deviation between *ϕ* and *ϕ*'. Our findings prove that by adjusting *ϕ*, it is possible to transport a desired degree of entanglement in 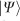 to a predefined *z*-position.

### Discussion

Our results highlight the fact that it is possible to engineer vectorial light fields whose degree of non-separability oscillates in free-space, from fully vector to scalar, as a function of the propagation distance, through spin–orbit coupling. While we have restricted ourselves to first-order vector vortex beams for the demonstration, the concept that we outline here is more general and can be applied to higher-order vector vortex modes as well as, in principle, any vector state with judicious choice of degree of freedom.

The surprising result is that our entanglement dynamics occur in free space under unitary conditions. We emphasize that while we have performed our experiments with coherent light for convenience, the same results are obtained for local entanglement of the internal degrees of freedom of a single photon. Neither theory nor experiments differentiate between these two cases, and thus, we address topical questions as to the notion of local and classical entanglement and its propagation dynamics.

An important aspect of this work is the practical approach to the generation and propagation of the fields. It is possible to engineer the desired effect using a Sagnac interferometer in which an input radially polarized vector beam is split into two beams traveling along each arm: one of the beams is switched to azimuthal polarization, with a half-wave plate, and interfered with the radially polarized beam. In the third arm, both beams propagate in opposite directions while bearing orthogonal states of polarization, and they thus generate a standing wave whose degree of entanglement varies along the propagation axis. This generating approach does not allow one to experimentally verify the spatially variant degree of entanglement. We offer a more powerful approach that utilizes digital generation and propagation enabled by an SLM. This approach allowed us to manipulate each vector beam independently and, among other options, perform digital propagation on each. Hence, both vector beams propagate in a collinear fashion in a manner that simulates propagation in opposite directions. This approach of generation and propagation enabled us to realize any state of 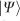 with a chosen degree of entanglement that can be adapted in real time by simply changing the displayed hologram.

**Figure 6:**
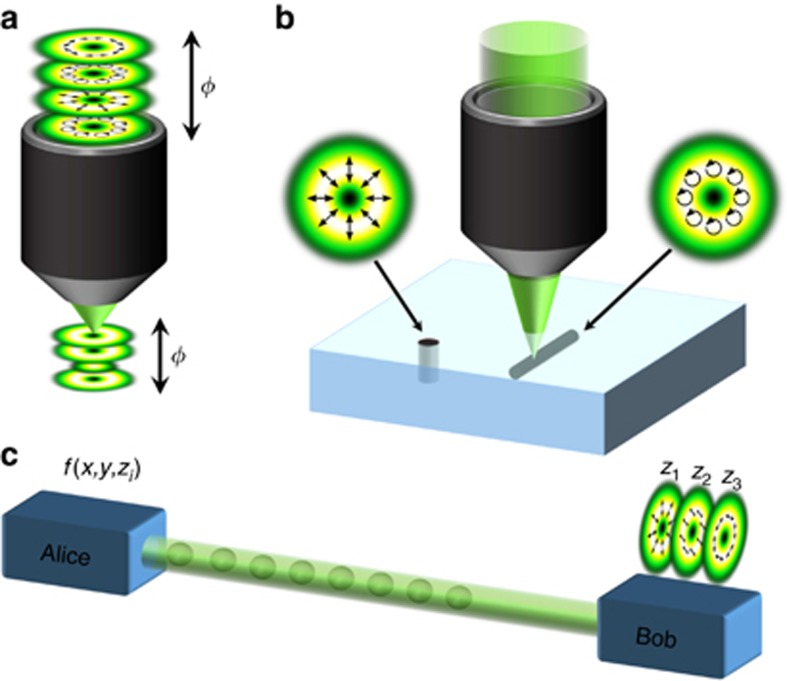
Exemplary applications of virtually counter-propagated, orthogonally polarized vector modes: (**a**) adjusting the mode at the focal region for, for example, STED microscopy systems, optical trapping or (**b**) laser material processing by digital propagation (phase shift *ϕ*), to create radially polarized beams for drilling and circularly polarized beams for cutting; (**c**) illustration of a novel quantum key distribution approach for the delivery of Alice's states to Bob.

Importantly, this approach allows us not only to monitor the degree of non-separability but also to provide on-demand specific states to the observer's positions. We believe that this method will enhance several noteworthy applications, namely, STED microscopy, optical trapping, quantum key distribution (QKD) and laser material processing systems, which we summarize graphically in Figure 6. For example, rapid changing of the mode type from circularly polarized light for cutting to radially polarized light for drilling would have clear benefits in processing materials with lasers^17, 18, 19^, while switching from a tight spot with radially polarized light to a donut beam with azimuthally polarized light (after an objective lens) is precisely the requirement for STED^31, 32^. In addition, the presented configuration paves the way for novel QKD approaches using a prepare-and-measure BB84 QKD protocol, with vector and scalar OAM modes as the orthogonal and mutually unbiased bases^47, 48^. This approach adds a new level of security to QKD protocols, since (as it has been noted to us) this work can be extended to a third dimension by considering the longitudinal mode function. Further, this tripartite description facilitates classical studies of GHZ-like states (see Supplementary Information), an exciting opportunity for further work with classically entangled states.

## Conclusions

We have demonstrated that by exploiting complex modes of light, it is possible to have an oscillating degree of local entanglement during propagation, even though the medium is considered to be unitary, that is, a medium in which the entanglement should not change. The result is a demonstration of spin–orbit coupling in paraxial light beams in free space. We have shown this effect with entangled internal degrees of freedom of polarization and spatial modes, and while our experiment was classical, the results hold equally well for local entanglement of the internal degrees of freedom of a single photon. In addition, we have demonstrated the concept behind the first tractor beam for local entanglement, which would be able to deliver a known degree of entanglement to some target plane. Our approach highlights intriguing questions about the notion of entanglement dynamics, opens a new topic in spin–orbit coupling and offers a new tool for a myriad of applications that would benefit from holographically controlled availability of vector and scalar states of light at the target plane.

## Author contributions

The experiments were performed by EO and CRG, with theoretical input from BN. All of the authors contributed to the data analysis, interpretation of the results and writing of the manuscript. AF conceived the idea and supervised the project.

## Figures and Tables

**Table 1:**
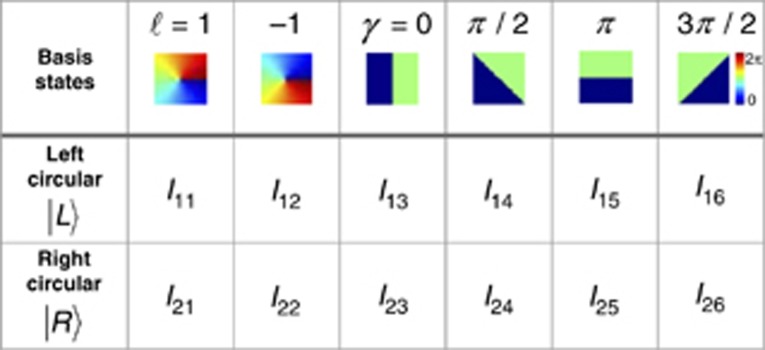
Normalized intensity measurements *I_uv_* for the determination of the expectation values 〈*σ_i_*〉
