# Supplementary material for: Entanglement beating in free space through spin–orbit coupling
Source: Light Sci Appl. 2018 May 4;7:18009–. doi: 10.1038/lsa.2018.9 (PMC6060074; doi:10.1038/lsa.2018.9)
Supplement: Supplementary Information [file lsa20189x1.pdf]

Supplementary information for:
Entanglement beating in free space through spin-orbit coupling

Eileen Otte,[1] Carmelo Rosales-Guzmán,[2,*] Bienvenu Ndagano,[2] Cornelia Denz,[1] and Andrew Forbes[2]

[1] *Institute of Applied Physics, University of Muenster, Corrensstr. 2/4, D-48149 Muenster, Germany*

[2] *School of Physics, University of the Witwatersrand, Private Bag 3, Wits 2050, South Africa*

Superposition of counter-propagating orthogonal vector fields – Theory

The realisation of the light field $|\Psi(x,y,z)\rangle$ with a spatially varying degree of entanglement $E(|\Psi\rangle, z)$ was obtained by combining two orthogonal vector beams VB_1 and VB_2 propagating in opposite z-directions. These vector modes represented by $|\Psi^+_{\mathrm{VB}_1}\rangle$ and $|\Psi^-_{\mathrm{VB}_2}\rangle$ are generated by setting $\alpha_{\mathrm{VB}_1} = 0$ and $\alpha_{\mathrm{VB}_2} = \pi/2$, respectively, with $\ell = \ell_1 = -\ell_2$ and $p = p_1 = p_2$. The resulting light field of the superposition can be written as

$$\begin{aligned}|\Psi\rangle &= \frac{1}{\sqrt{2}}\left(|\Psi^+_{\mathrm{VB}_1}\rangle + |\Psi^-_{\mathrm{VB}_2}\rangle\right)\\ &= \frac{1}{2}\left(|LG_p^{\ell}\rangle\,|R\rangle + |LG_p^{-\ell}\rangle\,|L\rangle\right)\cdot \mathrm{e}^{\mathrm{i}k_z z}\\ &+ \frac{1}{2}\left(\mathrm{e}^{\mathrm{i}\frac{\pi}{2}}\,|LG_p^{\ell}\rangle\,|R\rangle + \mathrm{e}^{-\mathrm{i}\frac{\pi}{2}}\,|LG_p^{-\ell}\rangle\,|L\rangle\right)\cdot \mathrm{e}^{-\mathrm{i}k_z z}.\end{aligned} \tag{S1}$$

Regrouping terms with same polarisation leads to

$$\begin{aligned}|\Psi\rangle =& \frac{1}{2}\left(\mathrm{e}^{\mathrm{i}k_z z} + \mathrm{i}\,\mathrm{e}^{-\mathrm{i}k_z z}\right)\,|LG_p^{\ell}\rangle|R\rangle\\ &+ \frac{1}{2}\left(\mathrm{e}^{\mathrm{i}k_z z} - \mathrm{i}\,\mathrm{e}^{-\mathrm{i}k_z z}\right)\,|LG_p^{-\ell}\rangle|L\rangle,\end{aligned} \tag{S2}$$

which is Eq. (6) of the main text.

A tripartite GHZ-like description

Interestingly, Eq. (S2) can also be written as a tripartite classically entangled GHZ-like state between longitudinal position, polarisation and transverse degrees of freedom [1]. For this, notice that the exponential terms in Eq. (S2) can be written as

$$\begin{aligned}\frac{1}{2}\left(\mathrm{e}^{\mathrm{i}k_z z} + \mathrm{i}\,\mathrm{e}^{-\mathrm{i}k_z z}\right) &= \mathrm{e}^{\mathrm{i}\pi/4}\cos(k_z z - \pi/4),\\ \frac{1}{2}\left(\mathrm{e}^{\mathrm{i}k_z z} - \mathrm{i}\,\mathrm{e}^{-\mathrm{i}k_z z}\right) &= \mathrm{e}^{3\mathrm{i}\pi/4}\sin(k_z z - \pi/4).\end{aligned} \tag{S3}$$

If we define Eq. S3 as,

$$\begin{aligned}\mathrm{e}^{\mathrm{i}\pi/4}\cos(k_z z - \pi/4) &\equiv \langle z|C\rangle\\ \mathrm{e}^{3\mathrm{i}\pi/4}\sin(k_z z - \pi/4) &\equiv \langle z|S\rangle,\end{aligned} \tag{S4}$$

where $|C\rangle$ and $|S\rangle$ are two orthogonal state vectors, equation (S2) now becomes

$$|\Psi\rangle = |C\rangle|LG_p^{\ell}\rangle|R\rangle + |S\rangle|LG_p^{-\ell}\rangle|L\rangle. \tag{S5}$$

In this representation, observation of a particular polarisation state can be done by projecting onto the operator $|z\rangle\langle z|$. Moreover, a maximally entangled state is obtained for all z values that satisfies $|\langle z|C\rangle| = |\langle z|S\rangle|$, namely $z = n\lambda/4$, $n \in \mathbb{N}$.

Non-separability in orthogonal superpositions of vector fields – Theory

The degree of non-separability of a vector field given by

$$|\Psi\rangle = \sqrt{a}\cdot|u_R\rangle|R\rangle + \sqrt{1-a}\cdot|u_L\rangle|L\rangle, \tag{S6}$$

can be computed as [2]

$$E(|\Psi\rangle) = -\left[a\cdot\log_2(a) + (1-a)\cdot\log_2(1-a)\right]. \tag{S7}$$

Comparing Eqs. (S2) and (S6) and considering that $|u_{R,L}\rangle = |LG_p^{\pm\ell}\rangle\cdot\mathrm{e}^{\mathrm{i}\zeta_{R,L}}$, one can see that

$$\begin{aligned}\sqrt{a}\cdot\mathrm{e}^{\mathrm{i}\zeta_R} =& \frac{1}{2}\left(\mathrm{e}^{\mathrm{i}k_z z} + \mathrm{i}\,\mathrm{e}^{-\mathrm{i}k_z z}\right)\\ =& \frac{1}{2}\left[\cos(k_z z) + \sin(k_z z)\right]\cdot(1+\mathrm{i}).\end{aligned} \tag{S8}$$

From which, $\zeta_R = \pi/4$ $(= -\zeta_L)$ and

$$\sqrt{a} = \left|\frac{1}{2}\left[\cos(k_z z) + \sin(k_z z)\right]\cdot(1+\mathrm{i})\right|, \tag{S9}$$

that is,

$$\begin{aligned}a &= \frac{1}{4}\left[\cos(k_z z) + \sin(k_z z)\right]^2\cdot 2\\ &= \frac{1}{2}[1 + \sin(2k_z z)].\end{aligned} \tag{S10}$$

Substitution of Eq. (S10) into Eq. (S7) yields

$$\begin{aligned}E(|\Psi\rangle, z) = -\frac{1}{2}[1+\sin(2k_z z)]\log_2\left\{\frac{1}{2}[1+\sin(2k_z z)]\right\} -\\ \frac{1}{2}[1-\sin(2k_z z)])\log_2\left\{\frac{1}{2}[1-\sin(2k_z z)]\right\},\end{aligned} \tag{S11}$$

which can be written as Eq (7) of the main text, namely

$$\begin{aligned}E(|\Psi\rangle, z) = 1 - \frac{1}{2}\left[1+\sin(2k_z z)\right]\cdot\log_2\left[1+\sin(2k_z z)\right] -\\ \frac{1}{2}\left[1-\sin(2k_z z)\right]\cdot\log_2\left[1-\sin(2k_z z)\right].\end{aligned} \tag{S12}$$

* Email: carmelo.rosalesguzman@wits.ac.za

Counter-propagating higher-order vector and scalar modes

The realisation of spatially varying degree of entanglement of the form $E(|\Psi\rangle, z)$ is not only facilitated by first-order vector modes with $\ell = 1$ and $p = 0$ but also by the application of higher-order modes [3] with $|\ell| > 1$ and $p > 0$. In this case, vector modes $|\Psi^+_{\mathrm{VB}_1}\rangle$ and $|\Psi^-_{\mathrm{VB}_2}\rangle$ still represent counter-propagating, orthogonal modes, whereby $\ell = \ell_1 = -\ell_2$ and $p = p_1 = p_2$ for both vector modes, as indicated within the theoretical description above. Further, an appropriate choice of $\alpha_{\mathrm{VB}_{1,2}}$ ensures the modes' orthogonality. If these requirements are fulfilled, different light fields $|\Psi\rangle$ can be created. Figure S1 sketches two examples of these fields with (a) $\ell = 2$, $p = 0$ and (b) $\ell = 2$, $p = 1$ ($\alpha_{\mathrm{VB}_1} = 0$, $\alpha_{\mathrm{VB}_2} = \pi/2$ for both cases). Here, different transverse planes of $|\Psi\rangle$ (normalised intensity + polarisation) are illustrated for chosen propagation distances z. The respective degree of entanglement E is visualized by the red curve between (a) and (b), whereby the arrow shows corresponding values of $k_z z+\varphi$ with $[0, \pi/2]$ and $[\pi/2, \pi]$ belonging to first and second line of (a) and (b). Initial vector modes propagating in $+z$- and $-z$-direction are indicated at the left and right edge, respectively. Note that, function $E(|\Psi\rangle, z)$ is independent of chosen mode numbers ℓ and p, even if other characteristics as intensity and polarisation of respective light field $|\Psi\rangle$ change according to ℓ and p.

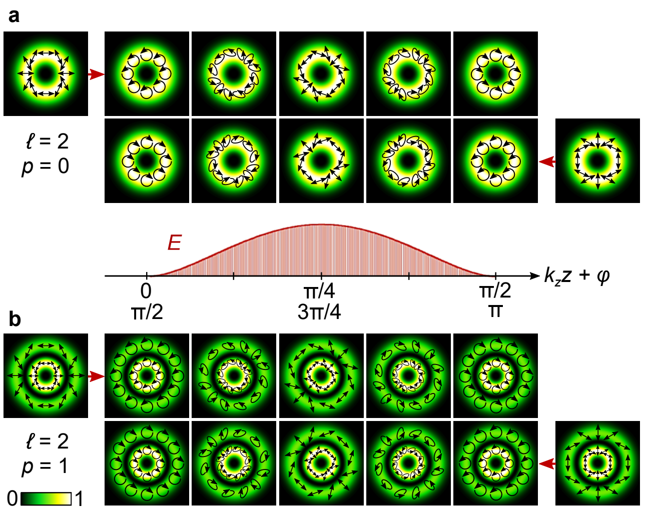

Figure S1: Space-variant degree of entanglement by counter-propagating vector modes of higher order with (a) $\ell = 2$, $p = 0$ and (b) $\ell = 2$, $p = 1$. Initial modes are shown at the right and left edge, whereby central images present different transverse planes (intensity + polarisation) within $|\Psi\rangle$. Respective degree of entanglement E depending on $k_z z + \varphi$ is depicted by red curve ($\varphi = -\pi/4$).

In contrast to counter-propagating, orthogonally polarised vector modes, scalar modes cannot be used to realise the spatially varying degree of entanglement. Considering two counter-propagating scalar modes $|\Psi^+_{\mathrm{sc},1}\rangle$

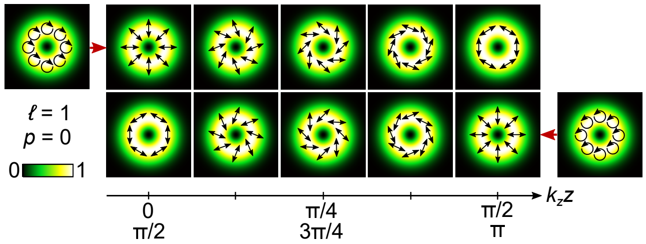

Figure S2: Counter-propagating scalar modes of opposite helical charge ($\ell = \pm 1$) and polarisation ($|R\rangle$, $|L\rangle$): Initial modes are shown at the right and left edge, whereby central images present different transverse planes (intensity + polarisation) within $|\Psi\rangle = \frac{1}{\sqrt{2}}\left(|\Psi^+_{\mathrm{sc},1}\rangle + |\Psi^-_{\mathrm{sc},2}\rangle\right)$ (first row belongs to $k_z z \in [0, \pi/2]$, second to $k_z z \in [\pi/2, \pi]$). Respective degree of entanglement is spatially constant with $E(|\Psi\rangle) = 1\,\forall z$.

and $|\Psi^-_{\mathrm{sc},2}\rangle$ of opposite helicity and circular polarisation, the resulting light field $|\Psi\rangle$ is represented by

$$\begin{aligned}|\Psi\rangle &= \frac{1}{\sqrt{2}}\left(|\Psi^+_{\mathrm{sc},1}\rangle + |\Psi^-_{\mathrm{sc},2}\rangle\right)\\ &= \frac{1}{\sqrt{2}}\left(\mathrm{e}^{\pm \mathrm{i}k_z z}|LG^{\ell}_{p}\rangle|R\rangle + \mathrm{e}^{\mp \mathrm{i}k_z z}|LG^{-\ell}_{p}\rangle|L\rangle\right)\\ &= \sqrt{a}|u_R\rangle|R\rangle + \sqrt{1-a}|u_L\rangle|L\rangle. \end{aligned} \tag{S13}$$

In this case, the spatial modes are given by $|u_R\rangle = \mathrm{e}^{\pm \mathrm{i}k_z z}|LG^{\ell}_{p}\rangle$ and $|u_L\rangle = \mathrm{e}^{\mp \mathrm{i}k_z z}|LG^{-\ell}_{p}\rangle$ with $\langle u_{L,R}|u_{R,L}\rangle = 0$. Consequently, the factor $a = 1/2$ is spatially independent resulting in a constant degree of entanglement of $E(|\Psi\rangle) = 1$ ($\forall z$). The respective light field for $\ell = 1$, $p = 0$ and $\varphi = 0$ is shown in Fig. S2. Here, in each z-plane a vector mode is realized.

Spin-orbit interaction

To calculate the spin and orbit components of our field, we express the total angular momentum in the z-direction as [4]

$$J_z = \frac{\int \mathrm{Im}\left\{\boldsymbol{E}^* \cdot \partial_\phi \boldsymbol{E} + \boldsymbol{e_z} \cdot \boldsymbol{E}^* \times \boldsymbol{E}\right\} d\boldsymbol{R}}{\int \boldsymbol{E}^* \cdot \boldsymbol{E} d\boldsymbol{R}} \tag{S14}$$

where the terms have their usual meaning. It is easy to show that this integral is zero for the $\ell = \pm 1$ subspace for the entire standing wave. We can calculate the spin and orbital components separately as

$$S_z \propto \int \mathrm{Im}\left\{E_x^* E_y - E_y^* E_x\right\} dA \tag{S15}$$

$$L_z \propto \int \mathrm{Im}\left\{E_x^* \partial_\phi E_x + E_y^* \partial_\phi E_y\right\} dA \tag{S16}$$

where here the x and y subscripts refer to the field components of the initial superposition but written in

the horizontal and vertical basis, i.e., $|\Psi\rangle = E_x\hat{\boldsymbol{x}} + E_y\hat{\boldsymbol{y}}$. After some algebra one can show that these terms become

$$E_x = \frac{1}{\sqrt{2}}\left[\mathrm{e}^{-\mathrm{i}k_z z}\cos(\ell\phi) - \mathrm{e}^{\mathrm{i}k_z z}\sin(\ell\phi)\right] \tag{S17}$$

and

$$E_y = \frac{1}{\sqrt{2}}\left[\mathrm{e}^{-\mathrm{i}k_z z}\sin(\ell\phi) - \mathrm{e}^{\mathrm{i}k_z z}\cos(\ell\phi)\right] \tag{S18}$$

which after substitution into the above, we find (after some simple algebra): $S_z \propto \sin(2k_z z)$ and $L_z \propto -|\ell|\sin(2k_z z)$. We have a sum that reflects a coupling between spin and orbit components, with one increasing as the other decreases: $J_z \propto (1-|\ell|)\sin(2k_z z)$. For the $\ell = \pm 1$ subspace the sum always adds to zero so that the total angular momentum is conserved through this SO coupling.

[1] Balthazar WF, Souza CER, Caetano DP, Galvão EF, Huguenin JAO, and Khoury AZ. Tripartite nonseparability in classical optics. *Opt. Lett.* 2016; **41**: 5797–5800.

[2] McLaren M, Konrad T, and Forbes A. Measuring the nonseparability of vector vortex beams. *Phys. Rev. A* 2015; **92**: 023833.

[3] Otte E, Alpmann C, and Denz C. Higher-order polarization singularitites in tailored vector beams. *J. Opt.* 2016; **18**:074012.

[4] Berry MV, Jeffrey MR, and Mansuripur M. Orbital and spin angular momentum in conical diffraction. *Journal of Optics A: Pure and Applied Optics* 2005; **7**: 685.
